# A combination of cognitive behavioral therapy and enteral nutrition support is beneficial for patients with severe acute pancreatitis in intensive care unit

**DOI:** 10.3389/fmed.2026.1721923

**Published:** 2026-03-02

**Authors:** Jingyi Chen, Kaibing Xiao, Xiangyu Tong, Xia Zhao

**Affiliations:** Department of Critical Care Medicine, The First Hospital of Guangyuan, Guangyuan, Sichuan, China

**Keywords:** cognitive behavioral therapy, enteral nutrition support, inflammatory factor, pain, quality of life, severe acute pancreatitis

## Abstract

This study aimed to assess the effects of cognitive behavioral therapy (CBT) combined with enteral nutrition support on patients with severe acute pancreatitis (SAP) in the intensive care unit (ICU). From May 2022 to August 2023, 50 SAP patients admitted to our hospital were randomly assigned to a control group (routine nursing) and a study group (CBT nursing based on routine nursing). Both groups received enteral nutrition support. After 14 days of intervention, the study group showed higher blood biochemical indicators (albumin, total protein, globulin) and lower inflammatory marker levels (procalcitonin, C-reactive protein, interleukin-6) compared to the control group. The study group also had shorter ICU and hospital stays, quicker resumption of oral feeding, lower pain scores, and higher quality of life scores. No significant differences in gastrointestinal adverse reactions and infections were observed between the two groups. We concluded that CBT combined with enteral nutrition support benefits SAP patients in the ICU by improving nutritional status, reducing inflammation, relieving pain, and enhancing quality of life.

## 1 Introduction

Pancreatitis, a primary cause of gastrointestinal disease-related hospitalizations, exhibits high mortality and severely impairs patients’ quality of life due to its potential to induce severe, recurrent pain and negatively affect physical, psychological, and social functioning (1). Acute pancreatitis (AP) manifests as sudden inflammatory attacks of the pancreas, with around 80% of cases presenting as mild edematous forms that resolve within days (2). However, approximately 20% progress to severe AP (SAP), marked by early or delayed systemic and local complications, and a mortality rate that can reach 50%, compared to 2%–5% across all AP forms (3).

In critical care, managing SAP patients is particularly challenging. They endure not only physical pain from acute pancreatic inflammation and complications but also significant psychological stress (4). In the intensive care unit (ICU), patients face life-threatening conditions, invasive procedures, constant monitoring, and isolation, all of which can trigger anxiety, depression, and fear. These emotions, mediated through the neuroendocrine system, adversely affect physiological functions, such as increasing stress hormone secretion (e.g., adrenaline), weakening immune responses, delaying wound healing, and elevating complication risks, ultimately impacting disease prognosis and rehabilitation (5). Therefore, addressing psychological issues in SAP patients through effective interventions is crucial for enhancing treatment outcomes and patient prognosis.

Cognitive-behavioral therapy (CBT) is a widely used method in the field of psychological therapy, aiming to change patients’ thoughts and behavioral patterns, helping them better control their symptoms and thereby reducing the impact of pain on their psychological and social health (6). Although previous studies mainly focused on psychological intervention for patients with chronic diseases (7), CBT’s applicability to acute diseases is also recognized. It has been reported to significantly reduce depressive symptoms in patients with acute post-traumatic stress disorder and improve their quality of life (8). CBT’s core lies in helping patients identify and change negative cognitive and behavioral patterns, regardless of whether the disease is chronic or acute (9). For SAP patients, CBT can correct erroneous perceptions and establish positive thinking patterns during the acute onset period. Implementing CBT in the ICU is feasible, as patients usually have clear consciousness and certain cognitive and communication abilities, and medical staff have established close contact with them.

Nutritional support therapy is an important part of comprehensive treatment for SAP patients. Due to their long-term high catabolic state, gastrointestinal absorption function is affected, leading to malnutrition and gastrointestinal dysfunction (10). Nutritional support therapy provides necessary nutrients, maintains intestinal function, protects the intestinal mucosal barrier, reduces gastrointestinal damage, improves immune function, and promotes recovery (11). Studies have shown that nutritional support therapy is effective in reducing SAP mortality (12). However, the impact of CBT combined with enteral nutrition support on SAP patients in the ICU remains unclear.

Therefore, this study assessed the influence of CBT combined with enteral nutrition support on SAP patients in the ICU.

## 2 Materials and methods

### 2.1 Study design

**FIGURE 1:**
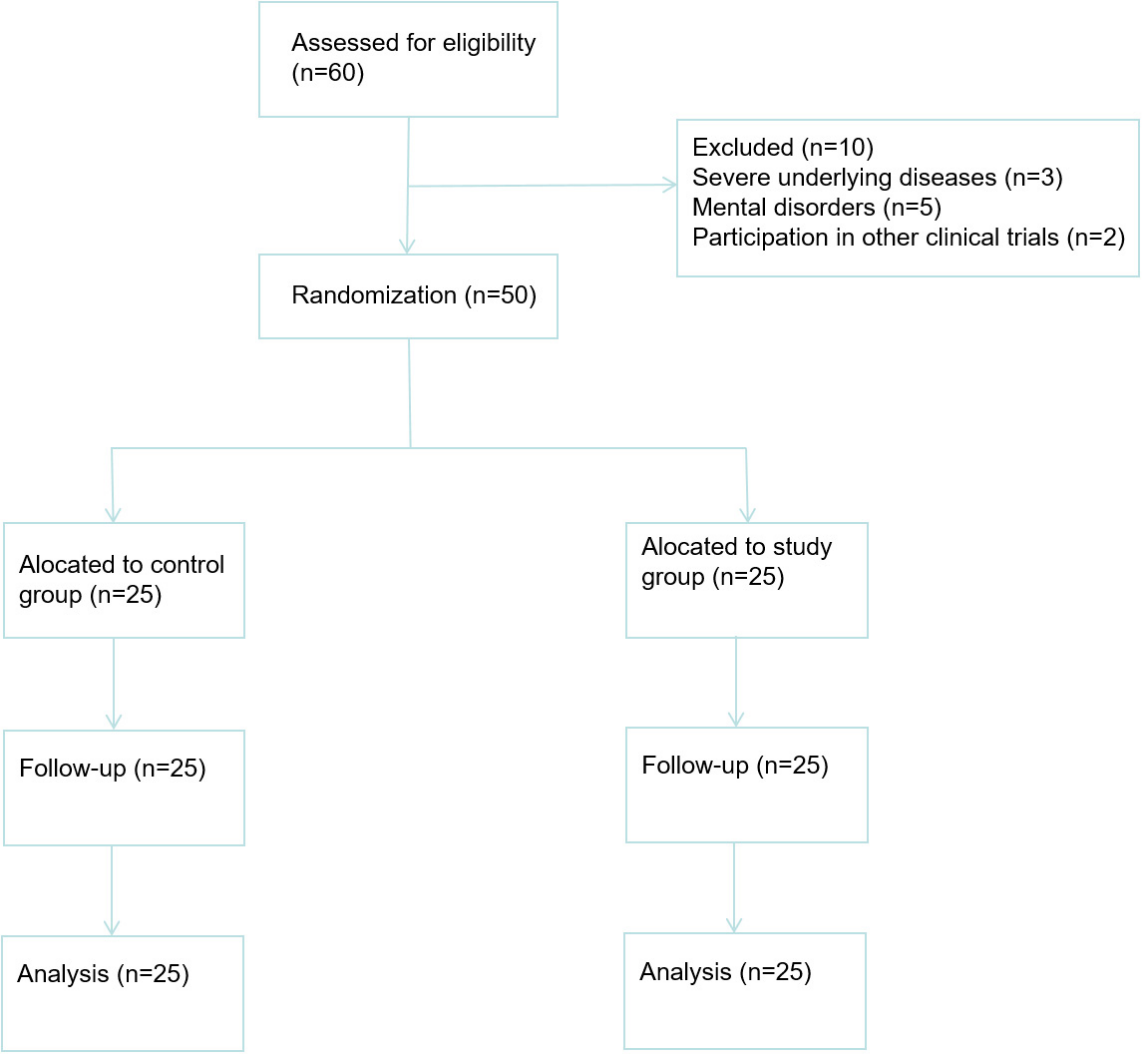
A CONSORT flow diagram.

This randomized controlled trial was conducted from May 2022 to August 2023. A total of 60 SAP patients were initially assessed for eligibility. Ten patients were excluded: 3 due to severe underlying diseases, five due to mental disorders, and two due to participation in other clinical trials. The remaining 50 patients were randomly assigned to the control group (*n* = 25) and the study group (*n* = 25) using a random number table. The CONSORT flow diagram is presented in Figure 1.

This study was approved by the Medical Ethics Committee of our hospital, and the patient’s family members signed informed consent. The research strictly adhered to the relevant requirements of the Consolidated Standards for Reporting Trials (CONSORT) and the Recommendations for Intervention Trials (SPIRIT) to ensure the scientificity, standardization and quality of the trial.

### 2.2 Inclusion and exclusion criteria

Inclusion criteria: (1) Patients were diagnosed with acute pancreatitis (AP) if they met at least two of the following conditions: Persistent abdominal pain; Serum amylase and/or lipase concentrations more than three times the upper limit of normal; Abdominal imaging consistent with AP changes. The diagnosis of AP strictly followed the Revised Atlanta Classification 2012 (13); (2) Patients met the diagnostic criteria for SAP, which was defined as persistent (lasting more than 48 h) organ dysfunction (14); (3) Aged between 18 and 90 years; (4) Nutritional risk screening 2002 (NRS 2002) score ≥ 3 points.

Exclusion criteria: (1) Contraindications for enteral nutrition preparations (complete intestinal obstruction, intestinal failure, intra-abdominal infection); (2) Shock, coma, unconsciousness; (3) Pregnancy or terminal illness; (4) Incomplete data; (5) Severe underlying diseases (severe heart, liver, and kidney dysfunction unrelated to AP); (6) Currently using other oral nutritional supplements or transferred to another hospital/department.

### 2.3 Sample size calculation

The sample size of 50 participants was determined based on recommendations for pilot randomized trials, allowing us to test feasibility indicators and initial therapeutic effects. With 25 participants per group, we could detect moderate (0.5) or significant (0.8) effects with a 90% confidence level and a two-sided significance level of 5% (15).

### 2.4 Randomization and allocation concealment

Random grouping was conducted using a random number table. Patients meeting inclusion criteria were sequentially numbered from 1 to 50 based on admission order. A random starting number was selected, and two-digit random numbers were read in sequence, corresponding to patient numbers. Patients with odd numbers were assigned to the control group, and those with even numbers to the study group. To ensure confidentiality, opaque sealed envelopes were used. A researcher not involved in recruitment wrote group information on paper, placed it in envelopes, and arranged them in patient number order. Another researcher opened envelopes sequentially to assign patients to groups.

### 2.5 Blinding

Due to the nature of the intervention, blinding of nursing staff and patients was difficult. However, a blind method was implemented for data collectors, who were unaware of group assignments and only responsible for collecting data according to a pre-designed form.

### 2.6 Enteral nutrition intervention

All patients were initiated on enteral nutrition (EN) within 48 h of admission. The route of administration was via nasogastric (NG) feeding using the product NUYRICIA (Wuxi, Jiangsu, China). Each patient received a daily energy intake of 25–30 kcal/kg/day delivered continuously over 24 h (16). The enteral nutrition formula contained maltodextrin, casein, vegetable oil, dietary fiber, minerals, vitamins, and trace elements.

During the first 1–3 days of EN initiation, patients underwent a low-dose and low-pump-rate treatment protocol. Specifically, on day 1, the initial dose was set at 10–15 kcal/kg/day with a pump rate of 20–30 mL/h. On day 2, the dose was increased to 15–20 kcal/kg/day with a pump rate of 30–40 mL/h. By day 3, the dose reached 20–25 kcal/kg/day with a pump rate of 40–50 mL/h. The dose was then gradually increased to the target of 25–30 kcal/kg/day by day 4, with a corresponding adjustment of the pump rate to ensure a smooth transition. The entire nutritional intervention lasted for 7 days. In terms of tolerance, patients were closely monitored for any signs of gastrointestinal complications. Abdominal distension was defined as an increase in abdominal girth of more than 2 cm from the baseline measurement, accompanied by patient-reported discomfort. Diarrhea was defined as the passage of three or more loose or liquid stools per day. During the 7-day EN period, in the control group, three patients (12%) experienced mild abdominal distension, which resolved spontaneously within 24–48 h without the need for intervention. Two patients (8%) in the control group developed diarrhea, which was managed with loperamide at a dose of 2 mg three times a day until the stools returned to normal. In the study group, two patients (8%) had mild abdominal distension, and one patient (4%) experienced diarrhea, which were also managed in a similar fashion as in the control group.

### 2.7 Nursing interventions

Control group: Received routine nursing, including vital sign monitoring, condition observation and recording, basic living care, and adherence to doctor’s instructions. Medical staff explained the treatment plan and provided emotional support and answers to questions within standard care.

Study group: Received CBT nursing intervention in addition to routine nursing, including:

#### 2.7.1 Frequency and duration

Overall intervention period: The CBT nursing intervention was carried out over a 2-week period.

Cognitive reconstruction: Sessions were held three times a week, with each session lasting approximately 45–60 min.

Behavioral activation: Medical staff met with patients four times a week to discuss and adjust behavioral goals and plans. Each meeting focused on behavioral activation lasted about 30–45 min. In addition, patients were encouraged to engage in positive behaviors daily as per the plan.

Relaxation training: Patients were guided to practice relaxation techniques twice a day, once during free time in the afternoon and once before sleep. Each practice session was 15–20 min long.

Communication and coordination with the medical team: A weekly meeting was held by the medical team, which lasted about 60–90 min.

#### 2.7.2 Provider training

All the nurses involved in delivering the CBT nursing intervention underwent a comprehensive training program before the start of the study. The training was conducted by experienced psychotherapists and consisted of both theoretical and practical components.

Theoretical training: Covered the basic principles and concepts of CBT, including the “cognition-emotion-behavior” triadic interaction model, behaviorist theory, and physiological and psychological principles underlying relaxation training. It also included an in - depth understanding of the core modules of CBT nursing intervention used in this study, such as cognitive reconstruction, behavioral activation, and relaxation training. The theoretical training lasted for two full days, with a total of 16 h of instruction.

Practical training: Involved role-playing exercises, case studies, and supervised practice sessions. Nurses practiced conducting cognitive reconstruction sessions, guiding behavioral activation, and demonstrating relaxation techniques under the supervision of psychotherapists. The practical training spanned 3 days, with 24 h of hands-on experience. After the training, nurses were required to pass a written test and a practical skills assessment to ensure they had mastered the necessary knowledge and skills for delivering the CBT nursing intervention.

#### 2.7.3 Core modules

Cognitive reconstruction: Based on the “cognition-emotion-behavior” triadic interaction model, cognitive reconstruction aimed to identify and correct incorrect cognitions, establish positive thinking patterns, and improve emotional states and behavioral performance. Medical staff communicated gently and patiently with patients, introduced themselves and the ICU environment, and established trust. They used questioning and listening to guide patients to express thoughts and feelings, identify automatic thoughts, and jointly assess their rationality and authenticity. Based on assessment results, medical staff helped patients correct erroneous perceptions and establish positive thinking patterns using methods like Socratic questioning and behavioral experiments. They reinforced new cognitions and encouraged patients to apply positive thinking patterns in daily life, consolidating them through regular follow-ups and health education.

Behavioral activation: Based on behaviorist theory, behavioral activation encouraged patients to increase participation in positive behaviors, breaking the vicious cycle of negative emotions and behaviors. Medical staff worked with patients to formulate specific, feasible, and measurable behavioral goals, developed detailed action plans, and provided support and encouragement during implementation. They observed patient reactions and physical conditions, making timely adjustments to the behavioral plan. When patients achieved goals, timely reinforcement and rewards were given to enhance motivation for continued positive behaviors.

Relaxation training: Based on physiological and psychological principles, relaxation training reduced the body’s stress response by consciously controlling physiological activities, achieving mind-body relaxation and alleviating anxiety and tension. Common methods included deep breathing relaxation, progressive muscle relaxation, and imagery relaxation. Medical staff selected appropriate methods based on patient conditions and preferences, explained steps and precautions, demonstrated techniques, guided practice, provided feedback and corrections, and encouraged daily practice for 15–20 min during free time or before sleep. They conducted regular follow-ups to understand practice situations and provide guidance and encouragement.

Communication and coordination with the medical team: A weekly meeting was held by a medical team consisting of doctors, nurses, and psychotherapists to discuss patient conditions, treatment plans, and psychological status. The psychotherapist reported on CBT implementation and patient psychological changes, the doctor introduced condition progress and treatment plan adjustments, and the nurse provided feedback on daily care and behavioral performance. Regular meetings strengthened information sharing and communication coordination, ensuring orderly cooperation between CBT and medical treatment. When abnormal conditions were discovered or treatment plans needed adjustment, medical staff promptly communicated with team members. Doctors, nurses, and psychotherapists jointly participated in ward rounds to achieve a comprehensive understanding of patient physical and psychological conditions, promptly identify problems, and formulate solutions, improving treatment effect and patient satisfaction.

#### 2.7.4 Adherence

To ensure patient adherence to the CBT nursing intervention, several measures were taken:

Patient education: At the beginning of the intervention, patients were provided with detailed information about the purpose, process, and expected benefits of each CBT module. This helped patients understand the importance of their active participation and increased their motivation to adhere to the intervention.

Family involvement: Whenever possible, family members were encouraged to participate in the intervention process. They were educated about the CBT techniques and were asked to support patients in practicing relaxation techniques at home and in encouraging positive behaviors.

Incentive system: A simple incentive system was established. Patients who showed good adherence to the intervention, such as consistently attending sessions, actively participating in discussions, and regularly practicing relaxation techniques, were given small rewards, such as a favorite book or a relaxation CD, to further motivate them.

We regularly monitored and recorded patients’ adherence to the intervention through attendance sheets, self-report questionnaires, and observations by medical staff. The overall adherence rate in the study group was 100%, indicating a relatively high level of patient engagement in the CBT nursing intervention.

The duration of the nursing intervention for both groups was 14 days.

### 2.8 Outcomes

#### 2.8.1 Primary outcomes

Blood biochemical parameters: Albumin (ALB), total protein (TP), hemoglobin (Hb), and globulin (GLB) were assessed before and 7 days after intervention.

Inflammatory markers: (PCT), C-reactive protein (CRP), and interleukin-6 (IL-6) were assessed before and 7 days after intervention.

Clinical outcomes: Length of stay in the ICU, length of hospital stay, and duration of resumption of oral eating.

### 2.8.2 Secondary outcomes

Gastrointestinal adverse events: Diarrhea, vomiting, constipation, abdominal pain, abdominal distension, gastric retention, and infection recorded within 7 days after intervention.

Pain: Assessed using the 24-h version of the brief pain scale short form within 7 days after intervention (17). Higher scores indicated greater pain interference and intensity.

Quality of life: Assessed using the Pancreatitis Quality of Life Instrument (PANQOLI) 14 days after administration (18). The higher the score, the better the quality of life.

### 2.9 Statistical analysis

Data were analyzed using SPSS 22.0 software. The Shapiro-Wilk test was used to measure the normality of the data distribution for each variable. For measurement data that met the assumption of normality, we used the independent-samples *t*-test for comparisons between two groups. The measurement data were expressed as mean ± standard deviation (x ± s). Effect size was displayed as 95% confidence interval (95% CI). Statistical data, which were presented as frequencies and percentages [*n* (%)], were analyzed using the chi-square (χ^2^) test. In addition, a two-way repeated-measures analysis of variance was used for multiple comparisons between the two groups. *P* < 0.05 was considered statistically significant.

## 3 Results

### 3.1 Comparison of general data

During the research period, 60 SAP patients were initially assessed. Ten patients were excluded: three due to severe underlying diseases, five due to mental disorders, and two due to participation in other clinical trials. A total of 50 patients met the inclusion criteria and agreed to participate. No patients were lost to follow-up, and all completed the corresponding nursing intervention and enteral nutrition treatment as per the research protocol. Baseline data of the two groups were comparable (*P* > 0.05, Table 1).

**TABLE 1:** General data of two groups.

Parameters	Control group	Study group	*P*-value
Gender (man/woman, cases)	11/14	13/12	0.571
Age (x ± s, years)	48.23 ± 14.32	50.11 ± 16.87	0.672
BMI	24.67 ± 3.15	25.24 ± 3.44	0.544
NRS 2002 score	4.36 ± 0.92	3.92 ± 0.91	0.095
APACHE II score	12.56 ± 3.42	11.87 ± 3.15	0.432
GCS score	13.48 ± 1.21	13.51 ± 1.23	0.931
Complications, cases			
Diabetes	4	6	0.725
Hypertension	6	5	>0.999
Hyperlipidemia	5	4	>0.999
Abdominal infection	8	9	>0.999
Types of organ failure			
Respiratory failure	3	4	>0.999
Renal failure	3	2	>0.999
Cardiovascular failure	2	4	>0.999
Duration of organ failure (days)	7.25 ± 1.36	7.37 ± 1.42	0.761
Etiology of acute pancreatitis			
Gallstones	8	9	>0.999
Alcohol	7	6	>0.999
Hypertriglyceridemia	4	5	>0.999
Trauma	2	3	>0.999
Idiopathic	4	2	>0.999
Analgesia			0.568
Opioids	10	12	–
Non-opioid analgesics	15	13	–
Ventilation			
Mechanical ventilation	4	3	>0.999
Non-invasive ventilation	3	2	>0.999
Antibiotics			0.757
Yes	18	17	–
No	7	8	–

BMI, body mass index; NRS 2002, nutritional risk screening 2002; APACHE II: Acute Physiology and Chronic Health Evaluation II; GCS: Glasgow Coma Scale.

### 3.2 Comparison of blood biochemical parameters

There were no significant differences in blood biochemical indicators between the two groups before intervention (*P* > 0.05). After 7 days of intervention, ALB, TP, and GLB levels in both groups were higher than before intervention (*P* < 0.05, 95% CI: −5.783 to −3.167; *P* < 0.05, 95% CI: −8.439 to −3.291; *P* < 0.05, 95% CI: −9.405 to −3.205). Compared with the control group, the study group had significantly higher ALB, TP, and GLB levels (*P* < 0.05, 95% CI: −2.873 to −0.166; *P* < 0.05, 95% CI: −5.559 to −0.411; *P* < 0.05, 95% CI: −6.585 to −0.385; Table 2).

**TABLE 2:** Comparison of blood biochemical parameters (g/L).

Groups	Before intervention		After intervention	
Parameters	Control group	Study group	Control group	Study group
ALB	30.08 ± 3.05	30.02 ± 3.01	33.02 ± 3.34*	36.12 ± 4.12*,^#^
TP	60.83 ± 6.15	60.77 ± 6.13	63.65 ± 6.38*	69.68 ± 7.21*,^#^
Hb	92.73 ± 17.23	90.85 ± 15.82	92.73 ± 17.23	94.18 ± 20.16
GLB	27.69 ± 7.15	28.35 ± 7.81	31.17 ± 8.02*	37.48 ± 8.21*,^#^

*P < 0.05 compared with before intervention.

^#^*P* < 0.05 compared with control group.

### 3.3 Comparison of proinflammatory markers

There were no significant differences in proinflammatory marker levels between the two groups before intervention (*P* > 0.05). After 7 days of intervention, PCT, CRP, and IL-6 levels in both groups were lower than before intervention (*P* < 0.05, 95% CI: 0.246–0.803; *P* < 0.05, 95% CI: 123.6–130.3; *P* < 0.05, 95% CI: 0.405–0.904). Compared with the control group, the study group had significantly lower PCT, CRP, and IL-6 levels (*P* < 0.05, 95% CI: 0.006–0.563; *P* < 0.05, 95% CI: 7.50–14.25; *P* < 0.05, 95% CI: 0.045–0.544; Table 3).

**TABLE 3:** Comparison of proinflammatory markers.

Groups	Before intervention		After intervention	
Parameters	Control group	Study group	Control group	Study group
PCT (ng/L)	1.23 ± 0.96	1.26 ± 1.02	1.02 ± 0.09*	0.42 ± 0.05*,^#^
CRP (mg/L)	168.32 ± 10.17	168.45 ± 12.13	52.35 ± 5.13*	30.74 ± 3.49*,^#^
IL-6 (ng/mL)	1.26 ± 0.85	1.38 ± 0.92	1.02 ± 0.08*	0.31 ± 0.05*,^#^

*P < 0.05 compared with before intervention.

^#^*P* < 0.05 compared with control group.

### 3.4 Comparison of clinical outcomes

The study group had shorter ICU and hospital stays and a shorter duration of resumption of oral eating than the control group (*P* < 0.001 and *P* = 0.004, respectively, Table 4).

**TABLE 4:** Comparison of clinical outcomes.

Outcome	Control group	Study group	*P*-value	95% CI
Length of stay in ICU (day)	14.21 ± 2.12	10.13 ± 1.66	<0.001	−5.164 to −2.996
Length of hospital stays (day)	27.63 ± 3.13	22.95 ± 2.56	<0.001	−6.308 to −3.052
Duration of resumption of oral eating (day)	16.74 ± 8.11	12.27 ± 4.45^#^	0.004	−8.190 to −0.750

### 3.5 Comparison of gastrointestinal adverse events as well as infection

There were no significant differences in gastrointestinal adverse events and infection between the two groups (*P* > 0.05, Table 5).

**TABLE 5:** Gastrointestinal adverse events and infection in two groups [*n*, (%)].

Adverse event	Control group	Study group	*P*-value
Diarrhea	5 (20%)	6 (24%)	0.732
Vomiting	2 (8%)	1 (4%)	>0.999
Constipation	1 (4%)	0 (0%)	>0.999
Abdominal pain	2 (8%)	1 (4%)	>0.999
Bloating	0 (0%)	2 (8%)	0.489
Stomach retention	1 (4%)	0 (0%)	>0.999
Infection	2 (8%)	1 (4%)	>0.999

### 3.6 Comparison of pain and quality of life

The study group had significantly less pain and higher quality of life scores than the control group (*P* < 0.05, Table 6).

**TABLE 6:** Pain and quality of life scores in two groups.

Variable	Control group	Study group	*P*-value	95% CI
Pain interference	4.34 ± 1.75	3.24 ± 1.03	0.011	−1.917 to −0.283
Pain intensity	4.36 ± 1.97	3.19 ± 1.23	0.024	−2.104 to −0.236
PANQOLI scores	53.16 ± 6.44	58.23 ± 6.21	0.006	1.472–8.668

## 4 Discussion

Our study explored the effects of CBT combined with enteral nutrition support on nutritional status, inflammatory response, clinical outcomes, incidence of gastrointestinal adverse events and infection, degree of pain, and quality of life in SAP patients in the ICU.

The results indicated that after 7 days of intervention, ALB, TP, and GLB levels in both groups were significantly higher than before intervention. Enteral nutrition support provides nutrients directly through the gastrointestinal tract, adjusting nutrient proportions and intake amounts according to patient requirements (19). High-quality proteins in enteral nutrition preparations, such as whey protein and casein, have high bioavailability and can be effectively absorbed and utilized, providing raw materials for liver synthesis of plasma proteins, thereby promoting increases in ALB, TP, and GLB levels (20). Moreover, the levels of ALB, TP, and GLB in the study group were higher than those in the control group, suggesting that CBT combined with enteral nutrition support can improve the nutritional status of SAP patients in the ICU. CBT and enteral nutrition support have a synergistic effect in improving nutritional status. Enteral nutrition support provides the material foundation, while CBT optimizes the environment for nutrient absorption and utilization from psychological and behavioral perspectives.

Our study also indicated that after 7 days of intervention, PCT, CRP, and IL-6 levels in both groups were significantly lower than before intervention. SAP patients are prone to intestinal mucosal damage, leading to disruption of the intestinal barrier function and translocation of bacteria and toxins, triggering systemic inflammatory response syndrome (SIRS) and aggravating the inflammatory reaction (11). Enteral nutrition support can provide nutritional substrates for intestinal mucosal cells, promoting proliferation and repair, enhancing tight junctions, maintaining intestinal barrier integrity, reducing bacteria and toxin translocation, and alleviating the systemic inflammatory response (21). Nutritional components in enteral nutrition can regulate immune cell activity, inhibiting pro-inflammatory cytokine production and release while promoting anti-inflammatory cytokine secretion (22). Furthermore, the levels of PCT, CRP, and IL-6 in the study group were lower than those in the control group, suggesting that CBT combined with enteral nutrition support can reduce the inflammatory response of SAP patients in the ICU. However, it is important to acknowledge a significant limitation in our interpretation of these results. While we have observed an association between the combined intervention and decreased levels of IL-6 and other inflammatory markers, we cannot claim a direct mechanistic link based on the current study design. Our study lacks direct evidence to precisely define how CBT interacts with the immune system and inflammatory pathways at a molecular or cellular level. To establish a more robust mechanistic understanding, future studies should incorporate more in-depth investigations.

Furthermore, our study indicated that the study group had shorter ICU and hospital stays, quicker resumption of oral feeding, less pain, and higher quality of life scores than the control group. SAP patients in the ICU often experience anxiety and depression due to disease severity and prognosis concerns, affecting appetite and willingness to eat. CBT, through psychological interventions, can alleviate anxiety and depression, enhance confidence, increase enthusiasm for eating, and encourage a normal diet (23). CBT can also help patients change their perception and evaluation of pain, reducing fear and anxiety about pain and lowering subjective pain perception. Relaxation training in CBT can help patients relax physically and mentally, reducing physical tension and alleviating pain. Similarly, it has been reported that CBT influences peripheral IL-6 in individuals with depression. In patients with symptomatic paroxysmal atrial fibrillation, online CBT leads to large improvements in the quality of life. In patients with acute ischemic stroke, CBT can effectively facilitate neurologic recovery, alleviate mental distress, and elevate health status (24).

In addition, our study indicated no significant differences in gastrointestinal adverse events and infection between the two groups, suggesting the safety and feasibility of the combination of CBT and enteral nutrition support for SAP patients.

### 4.1 Strengths and limitations

This study has certain advantages. It is the first to combine CBT with enteral nutrition support for SAP treatment, providing new ideas and methods. The research process was carried out strictly according to the established plan and evaluation indicators, ensuring standardization and data reliability. Patients included were representative of SAP patients in the ICU, and the results have reference value for clinical treatment.

However, there are some limitations. The sample size was relatively small, which may lead to insufficient stability of the results. A single-center design limits the external validity of the results. Single-blind operation may introduce subjective bias. The follow-up period was relatively short, preventing comprehensive observation of long-term effects.

Given these limitations, larger-scale, multi-center clinical trials are needed in the future to further verify the effectiveness and safety of CBT combined with enteral nutrition support. Double-blind or multi-blind methods can be adopted to reduce subjective bias. The follow-up period should be extended to comprehensively evaluate long-term effects. Additionally, the mechanism of action of CBT combined with enteral nutrition support can be further explored to provide more scientific theoretical basis for clinical treatment.

## 5 Conclusion

The results of this study indicate that combining CBT with enteral nutrition support has multiple positive effects on SAP patients. It improves nutritional status, reduces inflammation, relieves pain, and enhances quality of life. Therefore, this intervention measure has application potential in clinical practice and can be regarded as part of the comprehensive treatment for SAP patients.

## Data Availability

The datasets presented in this study can be found in online repositories. The names of the repository/repositories and accession number(s) can be found in this article/supplementary material.
